# Diverse Spectrum of Presentation of Coronary Slow Flow Phenomenon: A Concise Review of the Literature

**DOI:** 10.1155/2012/383181

**Published:** 2012-05-08

**Authors:** Muhammad A. Chaudhry, Marcus Smith, Elias B. Hanna, Ralph Lazzara

**Affiliations:** ^1^Section of Cardiology, Department of Medicine, The University of Oklahoma Health Sciences Center, Oklahoma City, OK 73126-0901, USA; ^2^Cardiovascular Section, Department of Medicine, Louisiana State University Health Sciences Center, New Orleans, LA 70112, USA

## Abstract

The coronary slow flow phenomenon (CSFP) is a disease entity characterized by slow progression of angiographic contrast in the coronary arteries in the absence of stenosis in the epicardial vessels. CSFP has a diverse presentation from mild chest discomfort to ST-segment elevation myocardial infarction. It can also have severe morbidity and mortality implications and can significantly hamper the quality of life of those affected. In this paper we present two patients with CSFP highlighting the diverse spectrum of presentation. A concise review of the literature is also provided emphasizing the epidemiology, pathogenesis, diagnostic parameters, treatment modalities, and clinical significance of this phenomenon.

## 1. Case Presentations

### 1.1. Patient 1

The first case involved a 43-year-old man with a past medical history of hyperlipidemia and hypertension who presented to the hospital with chest pain. The chest pain was described as a pressure-like sensation in the center of his chest, 8/10 in severity, and radiated down his left arm. Upon presentation, vital signs were stable, cardiac biomarkers were within normal limits, and the electrocardiogram (EKG) showed no ST/T-waves changes. Transthoracic echocardiogram showed normal left ventricle function and no wall motion abnormalities. Of note, the patient had a similar presentation and ER course two weeks prior.

**Figure 1:**
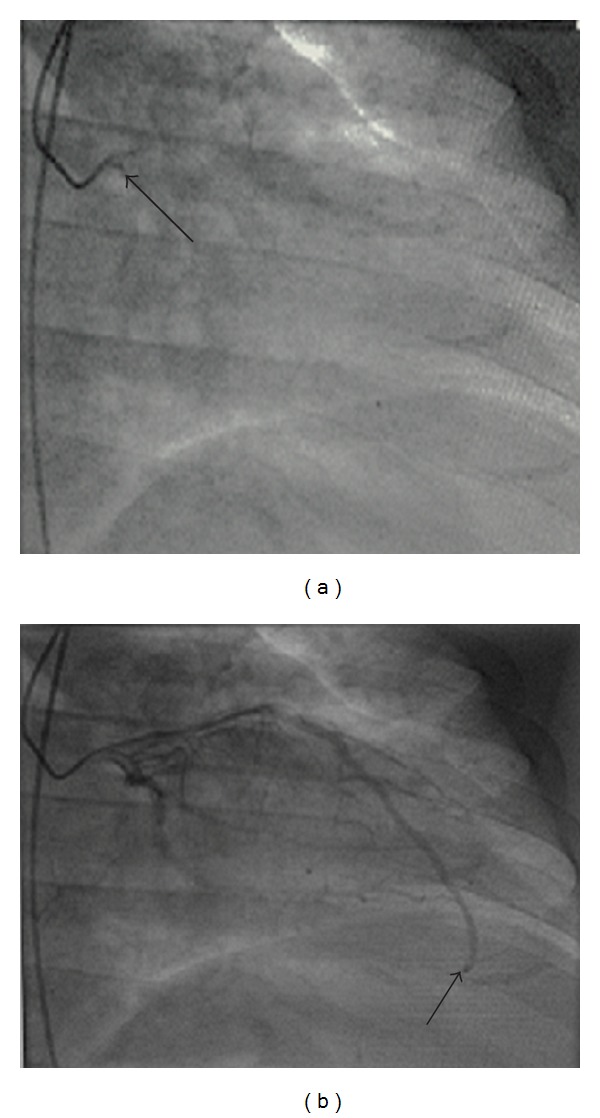
Corrected TIMI frame counting method of left anterior descending artery (LAD), Gibson's method with correction factor of 1.7 for LAD. Image on left (arrow) is when ostium is first opacified by contrast. Image on the right (arrow) is when the distal LAD is opacified by contrast. Patient's preadenosine TIMI frame count was 42 and postadenosine TIMI frame count was 14.

 Due to the fact that the patient continued to have 8/10 chest pain during his hospital stay, coronary angiogram was ultimately done. The angiogram showed normal coronary arteries without evidence of coronary vasospasm or an existing myocardial bridge. Slow flow, however, was noted in the left anterior descending artery (LAD). An intracoronary adenosine challenge was done and showed normalization of the TIMI (Thrombolysis in Myocardial Infarction) frame count (Figure 1). The patient was given the diagnosis of coronary slow flow phenomenon and started on dipyridamole 50 mg three times a day and discharged home.

 On six-month followup, the patient reported that he had been chest-pain-free. Shortly after his six-month followup, the patient ran out of his medicine and again began to experience chest pain. Once the patient was placed back on his dipyridamole, he became chest-pain-free.

### 1.2. Patient 2

**Figure 2:**
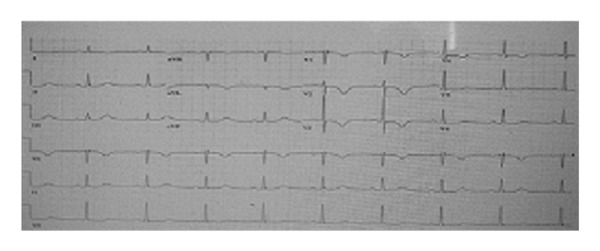
Electrocardiogram showing T-wave inversion in leads V1–V4. QT interval is also prolonged (QT-534 MS and QTc = 510 MS).

**Figure 3:**
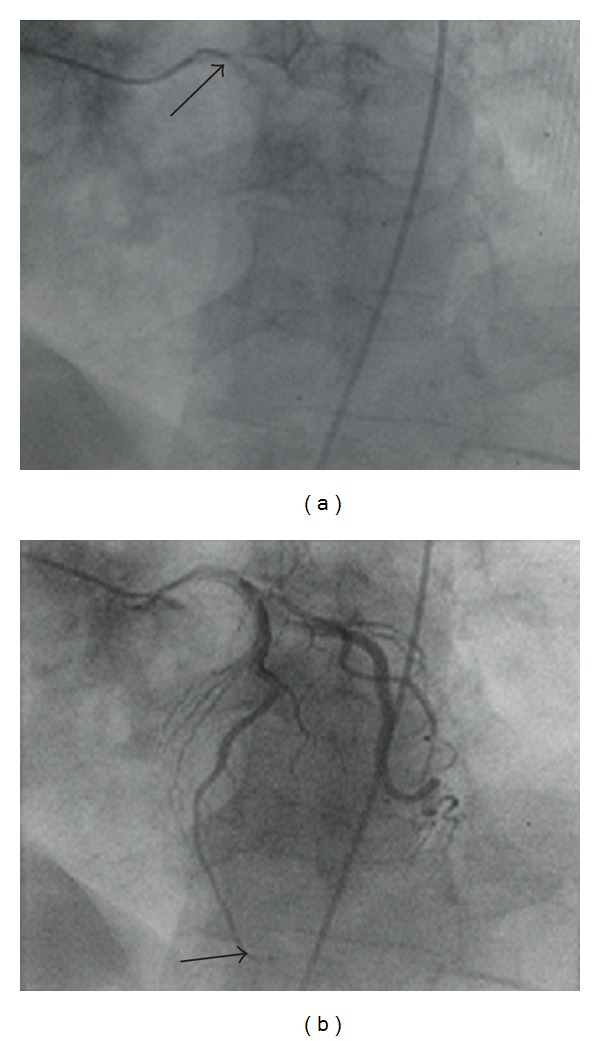
Corrected TIMI frame counting method of left anterior descending artery (LAD), Gibson's method with correction factor of 1.7 for LAD. Image on left (arrow) is when ostium is first opacified by contrast. Image on the right (arrow) is when the distal LAD is opacified by contrast. Patient's preadenosine TIMI frame count was 65 and postadenosine TIMI frame count was 16.

 The second case involved a 70-year-old gentleman with a past medical history of squamous cell carcinoma of the base of tongue six years prior. The patient presented with a single episode of chest pain at rest described as pressure-like and associated with shortness of breath and diaphoresis. Admission EKG revealed T-wave inversion in the anterior leads and a prolonged QT interval (Figure 2). Cardiac enzymes were also noted to be elevated (CKMB 8.3 IU/l and troponin I 1.14 ng/mL). Transthoracic echocardiogram showed normal left ventricular function with no wall motion abnormalities or left ventricular hypertrophy. A diagnosis of non-ST-elevation myocardial infarction (NSTEMI) was made, and the patient was started on the appropriate acute coronary syndrome medications. Left heart catheterization was done the following day and revealed normal coronary arteries with slow flow noted in LAD. An intracoronary adenosine challenge was done that showed normalization of the TIMI frame count (Figure 3). The patient was given the diagnosis of coronary slow flow and started on amlodipine 2.5 mg daily. On 6-month followup, the patient reports that he has been chest-pain-free.

## 2. Introduction

 CSFP was first described by Tambe et al. in 1972, and is defined as delayed opacification of coronary vessels during angiography without any evident obstructive disease [1]. Quantitatively, it is measured as increased Thrombolysis in Myocardial Infarction (TIMI) frame count. TIMI frame count, introduced by Gibson, is a reproducible index of coronary flow and represents the number of cine frames required for contrast to reach a prespecified distal coronary artery landmark. TIMI frame count is further corrected in the left anterior descending artery (LAD) by normalizing for the LAD length. The corrected TIMI frame count (CTFC) divides the absolute TIMI frame count in the LAD by 1.7. CSFP is defined as CTFC > 2 standard deviations from normal published range (21 ± 3) [2].

## 3. Presentation

 The presentation of this phenomenon is extremely diverse ranging from stable or unstable angina, non-ST-elevation myocardial infarction (NSTEMI), ST-elevation myocardial infarction (STEMI), nonsustained ventricular tachycardia (NSVT), and even vague chest discomfort. Yilmaz et al. found in their study that patients with CSFP had higher total cholesterol level (including higher low-density lipoprotein levels), higher body mass index, and a higher prevalence of metabolic syndrome [3]. Azzarelli et al. showed in a case series that patients with CSFP often present with recurrent chest pain [4]. New onset intermittent left bundle branch block (LBBB) has also been reported in association with CSFP in a patient presenting with acute coronary syndrome [5].

## 4. Incidence

 The overall incidence of CSFP is 1% among patients who undergo coronary angiography, especially those presenting with acute coronary syndrome [6]. In TIMI-IIIA study, the incidence of CSFP was 4% among patients who presented with unstable angina and had none or insignificant epicardial coronary artery disease [7].

## 5. Pathogenesis

The pathogenesis of CSFP is still not well understood. There are some typical histopathological features associated with CSFP based on which reduction of luminal diameter and functional obstruction are believed to be the key events in its pathogenesis. Mosseri et al. demonstrated medial hypertrophy, myointimal proliferation, and endothelial degeneration along with changes of myofibrillar degenerative foci with lipofuscin deposits at electron microscopic level [8]. Luminal narrowing was attributed to endothelial swelling and degeneration. Mangieri et al. substantiated these findings in CSFP patients by showing small vessel thickening with associated luminal narrowing, dilated interstitial spaces filled with granular fibrillar material, decreased intracellular glycogen, distorted mitochondrial cristae, and patchy myofibrillar disarray at electron microscopic level [9]. Based on this, it was suggested that fibromuscular hyperplasia and medial hypertrophy with consequent decrease in luminal diameter lead to functional obstruction and CSFP.

 The occurrence of CSFP is also believed to be strongly associated with elevated homocysteine levels which cause significant injury and consequent endothelial impairment [10]. Endothelial damage secondary to decreased nitric oxide levels, with elevated levels of nitric oxide synthase inhibitor and asymmetric dimethyl arginine (ADMA) are also considered as chief contributory factors [11, 12]. The basic underlying event is endothelial injury which leads to functional obstruction and disease manifestation.

 Aortic pulse pressure and pulsatility index was found to be significantly elevated in patients with CSFP. It is believed that this could possibly be caused by endothelial dysfunction in CSFP. Increased aortic stiffness in CSFP was shown by Tanriverdi et al. in a study of 154 patients which included 81 patients with angiographically proven CSFP and 73 patients with normal coronary flow [13].

 Impaired coronary flow reserve is another important feature in CSFP which is related to increased resting coronary microvascular tone. With increased myocardial oxygen demand, the inability to maximize coronary flow can reflect as persistent and recurrent chest pain in CSFP. In a study by Erdogan et al. which included 20 patients with CSFP and 15 controls, coronary flow reserve was significantly reduced in the CSFP group [18]. This was also shown by Mangieri et al. in a study of twenty-eight patients with CSFP in which 14 patients had coronary slow flow in one vessel and 14 had coronary slow flow in three vessels. After dipyridamole infusion, coronary angiography was done and single photon emission computed tomography (SPECT) scan was performed. Interestingly, TIMI frame count decreased after dipyridamole infusion in both groups while SPECT score increased in the first group and was reduced in the second group [19]. This suggests that myocardial perfusion in more extensive CSFP is significantly impaired and reflects the poor coronary flow reserve in these patients. A list of characteristic disease associations of CSFP is outlined in Table 1.

## 6. Treatment

 The treatment for CSFP is still a matter of debate and a number of drugs have shown variable benefits. Simvastatin has shown to improve myocardial perfusion in patients with CSFP [20]. The pleiotropic effect, direct endothelial function effect, and the antithrombotic and anti-inflammatory actions of statins appear to be responsible for this improved perfusion [21]. In a study by Fan et al. which included 91 patients with CSFP, significant improvement in coronary flow reserve was shown after treatment with atorvastatin 20 mg daily for eight weeks [22]. This study highlights the importance of statin therapy in treatment of CSFP.

 Nebivolol has also shown to be effective at improving endothelial function in patients with CSFP. It controls chest pain, decreases C reactive protein (CRP), and significantly improves coronary flow [23].

 Dipyridamole, which has a vasodilator effect on the coronary microvasculature, is being used as treatment for CSFP as well. In a study where 25 patients with CSFP were administered dipyridamole 75 mg three times a day for one month, coronary flow returned to normal in 70 out of 75 vessels. Typical angina complaints resolved in 17 patients (68%), and decreased in frequency in the remaining 8 patients (32%) [24]. Dipyridamole abolishes functional obstruction in coronary arteries with diameters less than 200 micrometers and is considered far superior in treating CSFP as compared to nitroglycerine [24].

 There is no substantial data regarding the use of conventional calcium L-channel blockers such as amlodipine in patients with CSFP. It was used in the second case as described above because calcium channel blockers are macro- and microvascular vasodilators used for slow flow during percutaneous coronary intervention (PCI). In one study regarding clinical and angiographic benefits of the calcium T-channel blocker mibefradil in CSFP patients, results showed reduction of total angina frequency by 56%, prolonged angina episodes > 20 minutes by 74%, and sublingual nitroglycerine consumption by 59% over a 12-month period. Angiographically, 13 out of 18 vessels showing TIMI-2 flow had abolition of the slow flow [25]. Currently, its use is limited due to occurrence of extensive drug interactions by inhibition of the cytochrome P450 3A4 pathway [26].

## 7. Syndrome X

 An important differential to be considered in the diagnosis of CSFP is coronary syndrome X. Over the last few years, a number of important distinguishing features between these two seemingly similar entities have been identified (Table 2). In a study of 49 patients, Atak et al. documented increased QTc dispersion in patients with CSFP, which greatly increases the predisposition of sudden cardiac death due to malignant ventricular arrhythmias [27]. CSFP has also been termed as syndrome Y because of its interesting occurrence in syndrome X patients, after administration of neuropeptide Y [28].

## 8. Prognosis

 The major long-term problem in CSFP patients is persistent and recurrent chest pain. One-third of these patients require readmission for an acute exacerbation [32]. In a study of 88 patients with angina and normal coronary arteries over a followup period of 6–11 years, chest pain diminished in 47%, and symptoms were unchanged in 24% or even worse in 29% of patients. However, 81% of patients with documented CSFP at coronary angiography reported chest pain to be similar or even worse than the initial episode [33].

Resting EKG abnormalities and positive exercise stress tests are more frequent in patients with CSFP. In one study of 53 patients with angina and angiographically normal epicardial coronary arteries and 40% having documented CSFP, 57% of patients had worsening or persistence of symptoms, and nonsignificant resting EKG abnormalities were seen in 27% while exercise-induced functional and perfusion abnormalities occurred in 69% of patients [34].

Patients with CSFP have a favorable long-term prognosis as reported by Lichtlen et al. in a prospective study of 176 patients with angina and normal coronary arteries. The median followup time period was 12.4 years. The rate of fatal myocardial infarction was found to be 0.05% per year, risk of death from coronary artery disease was 0.09% per year, and the rate of nonfatal myocardial infarction occurrence was 0.18% per year [35].

## 9. Conclusion

The major implications of this disease entity are reflected in the hampering of day-to-day life of CSFP patients with persistent chest pain and multiple hospital admissions. This is highlighted particularly well in the two cases presented here. There is a need for further extensive studies regarding the detailed pathogenesis and newer effective treatment modalities for this unique phenomenon with the potential to ameliorate the poor quality of life of patients with CSFP.

## Figures and Tables

**Table 1:** Characteristic disease associations of CSFP.

Salient associations of CSFP	
Chronically elevated resting microvascular endothelial tone [14]	
High incidence of metabolic syndrome [15]	
Normal large artery stiffness between acute episodes [16]	
Prolonged P-wave duration	
Increased P-wave dispersion [17]	
Increased aortic pulse pressure, fractional pulse pressure, and pulsatility index (parameters of aortic elasticity indicating increased aortic stiffness)	
Impaired coronary flow reserve	

**Table 2:** Comparison of coronary slow flow phenomenon (CSFP) versus syndrome X.

CSFP	Syndrome X
Males [29]	Postmenopausal females [29]
Current smokers [29]	Current or past smokers [29]
Unstable angina [30]	Stress-related angina [30]
Elevated resting microvascular tone [31]	Normal resting microvascular tone [31]
Normal vasodilator response to papaverine, adenosine, and during exercise [31]	Typically absent [31]
Greater rate of life threatening cardiac arrhythmias and sudden cardiac death [27]	Lower incidence as compared to CSFP [27]
